# Pathology of Ewing’s sarcoma/PNET: Current opinion and emerging concepts

**DOI:** 10.4103/0019-5413.69304

**Published:** 2010

**Authors:** Saral S Desai, Nirmala A Jambhekar

**Affiliations:** Department of Pathology, Tata Memorial Hospital, Dr. Ernest Borges Road, Parel, Mumbai, Maharashtra, India

**Keywords:** Ewing’s sarcoma, EWS-FLI1 translocation, pathology, PNET

## Abstract

Ewing’s sarcoma/PNET are small round cell tumors showing a varying degree of neuroectodermal differentiation. They are one of the commonest tumors of childhood and occur in bone and within soft tissues. Traditionally, light microscopy with the aid of immunohistochemical stains was suitable for diagnosis. But now translocation analyses are being used not only for the diagnosis and classification of small round cell tumors, but to ascertain their prognostic significance, detect micrometastasis, and monitor minimal residual disease, with potential for targeted therapy. This article analyzes the pathology, biology, and molecular aspects of Ewing’s sarcoma/PNET and discusses their clinical and therapeutic implications.

## INTRODUCTION

In 1918, Arthur Purdy Stout described a tumor composed of small round cells with rosettes, in the ulnar nerve,^1^ which came to be known as primitive neuroectodermal tumor (PNET). Later, James Ewing described a tumor of long bones composed of undifferentiated cells, which was radiosensitive^2^ (Ewing’s sarcoma). Over the years, these two tumors were described at various sites as two distinct entities. The distinction between these two tumors began to blur when Angervall and Enzinger (1975) described “an extraskeletal neoplasm resembling Ewing’s sarcoma”^3^ and Jaffe et al. published an article on “the neuroectodermal tumour of bone” in 1984.^4^ We now know that both Ewing’s sarcoma and PNET show similar translocations and are considered to be the ends of a histological spectrum of “Ewing’s family of tumors” (EFT). In the past two decades, our knowledge about the molecular events responsible for the development and progression of EFT has increased dramatically. Numerous technological developments have contributed to this greater understanding of cell biology and have shed light on the molecular mechanisms of malignant transformation. The analysis of these tumors by various molecular techniques may allow us not only to understand the biology of these lesions better but also to develop better techniques for their diagnosis and potential treatment.

## EPIDEMIOLOGY

EFT comprises 5–10% of primary bone tumors and is the second most common tumor in childhood.^5^ It occurs predominantly in children and young adults and shows a slight predilection for males.^6^ It has been described in siblings,^7^ though this is extremely rare and EFT is not a part of familial cancer syndromes.

### Sites of involvement and radiology

EFT usually arises from the diaphysis or metadiaphyseal region of long bones. It also arises from the pelvic bones and ribs. The other less-frequent and rare locations are the skull bones, the vertebra, the scapula, and the small bones of hands and feet. Any soft tissue site can be affected. The radiological findings are essential for making a histopathological diagnosis of any bone tumor. Ewing’s sarcoma involves the diaphysis of the bones and shows a permeative pattern of involvement with periosteal reaction.

### Tissue for pathological examination

A biopsy of the tumor is the best mode of obtaining a diagnosis. A core biopsy would usually suffice for making histological diagnosis and an open biopsy is only required when repeated attempts at obtaining adequate tissue with core biopsy have failed (technical problems, sclerotic bone, and previously treated case). If the representativeness of the biopsy is an issue, a frozen section examination can be performed for adequacy. However, the frozen section examination should be reserved for only selective cases, as freezing the tissue can distort the morphology and also lead to loss of antigens. The tumor tissue obtained should be fixed in 10% formalin. If the tissue has not been fixed adequately, it hampers histological examination and may lead to loss of antigens, which renders immunohistochemistry inconclusive. An inadequately fixed tissue would also cause autolysis and degeneration of DNA, making the material unsuitable for molecular analysis. The same is true for excision specimens. If the pathology laboratory is not in the same premises and the specimen cannot be transferred quickly, the excision specimen should be fixed in 10% formalin and the ratio of specimen to the amount of formalin should be atleast 1:10.

Fine needle aspiration cytology is not recommended for diagnosis as the amount of cells obtained is less and the material may not be adequate for immunohistochemistry and molecular analysis. The role of cytology should be confined in confirming metastasis or recurrence of tumor. Some tumor tissue at the time of biopsy can also be frozen for cytogenetic and molecular studies.

## HISTOLOGY

**Figure 1:**
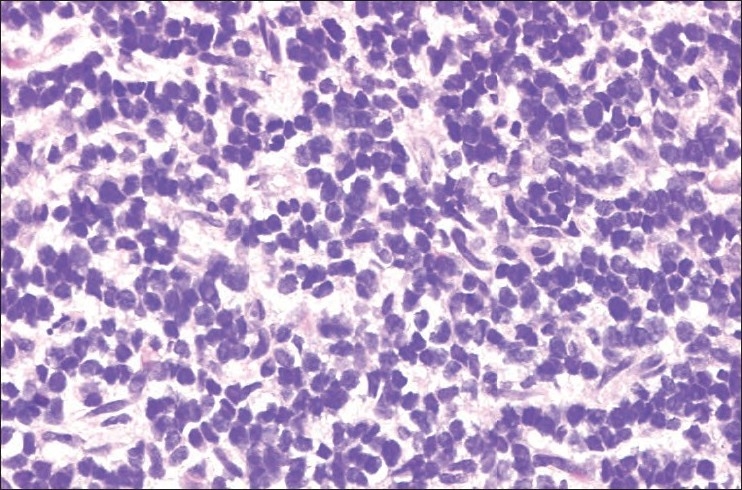
ES/PNET composed of sheets of small round blue cells

Ewing’s sarcoma/PNET is a prototype of the “small round cell” tumor group. It is composed of sheets of small cells with high nuclear to cytoplasmic ratio. The cytoplasm is scant, eosinophilic, and usually contains glycogen, which is detected by periodic acid Schiff stain and is diastase degradable. The nuclei are round, with finely dispersed chromatin, and one or more tiny nucleoli [Figure 1]. Occasional rosette formation is also seen. EFT does not produce any matrix. This tumor frequently undergoes necrosis and the residual viable cells show a “peritheliomatous” or a perivascular distribution. Rarely, EFT tumor cells can be large with irregular nuclear membrane and prominent nucleoli.^8^

**Figure 2:**
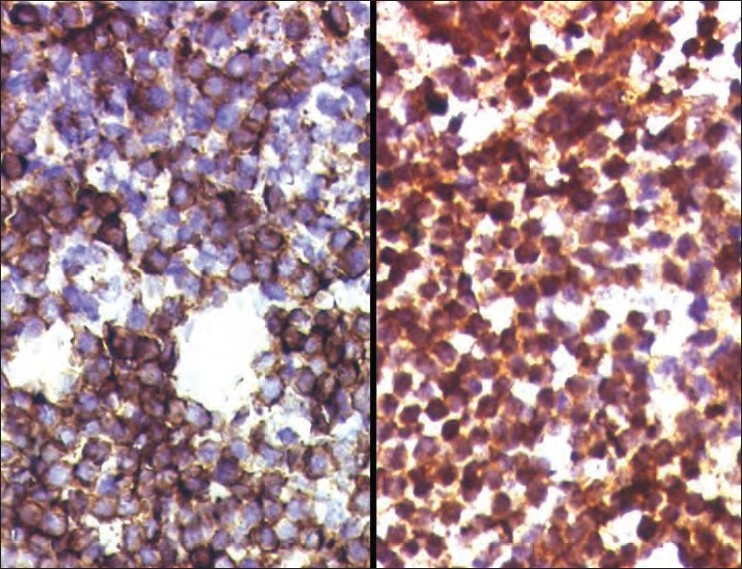
The tumor cells of EFT show membranous expression of CD99/MIC2 (left), and nuclear positivity for antibodies against FLI1 (right)

EFT cells show membranous expression of CD99 or MIC2 on immunohistochemistry [Figure 2].^9^ Antibody against FLI1, which is centered in the nucleus of the tumor cells [Figure 2], has been shown to be specific for EFT.^10^ Depending on the degree of neuroectodermal differentiation, the tumor cells may also express neuron-specific enolase (NSE), synaptophysin, and S-100 protein.

Immunohistochemistry is essential as the family of small round cell tumors is rather large and includes non-Hodgkin lymphoma, neuroblastoma, rhabdomyosarcoma, mesenchymal chondrosarcoma, retinoblastoma (Rb), and desmoplastic small round cell tumor (DSRCT). Other tumors can also show small round cells and these are osteosarcoma, synovial sarcoma, malignant peripheral nerve sheath tumor, and melanoma.

Although CD99 shows crisp and strong membrane positivity in EFT, it can also be positive in other tumors viz. lymphoblastic lymphoma, rhabdomyosarcoma, synovial sarcoma, mesenchymal chondrosarcoma, blastemal component of Wilms tumor, and rarely in DSRCT.^11^ Hence, a panel of immunohistochemical stains is employed to arrive at a definitive diagnosis. As stated above, CD99, FLI1, and NSE would be positive in ES/PNET. Non-Hodgkin lymphoma would express the lymphoid markers, i.e., CD45RB, CD3, CD20, and TdT; neuroblastoma would be positive for neuroendocrine markers (synaptophysin, chromogranin); rhabdomyosarcoma would be positive for skeletal muscle markers viz., desmin, myogenin, myo-D1, and myoglobin; and synovial sarcoma would also express pancytokeratins, EMA, BCL2, and calponin.

## MOLECULAR GENETICS

The EFT in 85% of cases is associated with translocation t(11;22)(q24;q12). This fusion of EWS gene on 22q12 with the FLI1 gene on 11q24 results in a chimeric fusion transcript EWS-FLI1.^12^ Type 1 (exon 7 of EWS to exon 6 of FLI1) and type 2 (exon 7 of EWS to exon 5 of FLI1) are the two types of typical translocation sites. In another 10–15% of cases, the translocation t(21;12)(22;12) resulting in EWS-ERG (Ets-related gene) fusion^13^ is seen. The remainder of 1–5% of the cases shows translocations, which involve fusion of EWS gene and a member of ETS family of transcription factors. The resulting translocations are EWS and ETV1 (Ets variant 1) (t(2;22)(p22;q12)),^13^ EWS and E1AF (Ets variant 4 – ETV4/E1A enhancer binding protein) (t(17;22)(q21;q12)),^14^ and EWS and FEV (t(2;22)(q33;q12)).^15^ More complex translocations have also been described.

Translocations involving EWS gene are observed in other tumors. EWS is fused to ATF1 (activating transcription factor 1) in malignant melanoma of soft parts, WT1 (Wilms tumor 1) in intra-abdominal DSRCT, CHOP in myxoid liposarcoma, and CHN in myxoid chondrosarcoma. In addition, EWS-like gene, TLS/FUS, is involved in tumor-associated gene fusions in myxoid liposarcoma and acute myeloid leukemia.

### The EWS gene

The EWS gene is a part of TET family of genes. Based on their structure and ability to bind RNA, TET proteins are thought to participate in transcription and RNA processing.^16^ In addition, EWS also interacts with splicing proteins and modulates splicing.^17^ One hypothesis that has been put forth is that TET proteins may provide a link between transcription and mRNA processing by binding components of both the transcription and splicing machinery.

### The FLI1 gene

The FLI1 gene was identified as the site of insertion of Friend’s murine leukemia virus.^18^ FLI1 is expressed in the hematopoietic and endothelial cells and in the mesenchymal cells of neural crest origin during embryonal development.^19^ FLI1 plays an important physiological role in hematopoiesis and vasculogenesis. Overexpression of FLI1 is observed to promote self-renewal,^20^ repress Rb protein,^21^ and induce BCL2 expression in erythroid cells with a corresponding enhancement of cell survival.^20^

### The effect of EWS-FLI1 expression in tumor development

In immunocompromised mice, the expression of EWS-FLI1 in murine NIH-3T3 cells resulted in anchorage independent growth and accelerated tumorigenesis with a tumor phenotype reminiscent of human Ewing’s sarcoma.^22^ These observations support the notion that EWS-FLI1 can stimulate oncogenesis and it is to a large extent responsible for the histological characteristics associated with EFT. Moreover, the expression of EWS-FLI1 in non-EFT tumor cells, e.g., neuroblastoma and alveolar rhabdomyosarcoma cells, resulted in transdifferentiation with the appearance of Ewing’s sarcoma features, including neural marker expression.^23^,^24^

Growth inhibitory effects of dominant negative FLI1 on Ewing’s sarcoma cell lines support the belief that EWS-FLI1 is involved in EFT development.^25^ Studies have demonstrated that antisense EWS-FLI1 and EWS-FLI1 siRNA expression in human Ewing’s sarcoma cell lines results in decreased cell growth *in vitro* and tumorigenicity in *in vivo*.^26^

### Mechanism of action of EWS-FLI1

EWS-FLI1 may participate in Ewing’s sarcoma pathogenesis by promoting at least two sets of events that synergize in tumor development and progression: cell proliferation and survival, by inducing among other candidate genes, PDGFC,^27^ insulin-like growth factor 1 (IGF-1),^28^ MYC,^29^ CCND-1^30^ and NKX2-2,^31^ and escape from apoptosis and growth inhibition, by repressing p21,^32^ p57^kip^,^33^ TGFβRII,^34^ and IGFBP3.^35^ In addition, EWS-FLI1 appears to play a critical role in inducing the EFT small round cell phenotype.

### Cell of origin

As EFT is a poorly differentiated tumor with both mesenchymal and neuroectodermal histological and immunohistochemical features, it is unclear if this tumor is of mesenchymal or neuroectodermal origin. Various experiments were undertaken and EWS-FLI1 introduced into fibroblasts, but EFT-like tumourigenesis did not take place. Instead, it led to growth arrest and apoptosis.^36^ EWS-FLI1 fusion gene, with the help of retrovirus, was introduced into murine cells with variable differentiation potential, ranging from embryonic stem cells and primary mesenchymal progenitor cells to embryonic fibroblasts. At the protein level, bone marrow derived mesenchymal progenitor cells maintained EWS-FLI1 expression,^37^ but not the embryonic fibroblasts and stem cells. When these cells were introduced into mice, a tumor composed of sheets of small round cells was formed. These small round cells expressed NSE and CD99 on immunohistochemistry and showed corresponding upregulation and downregulation of genes associated with EFT. These tumors also displayed high sensitivity to IGF-1R (insulin-like growth factor 1 receptor) inhibition, a hallmark of Ewing’s sarcoma.^38^ The age of development of Ewing’s sarcoma coincides with increased IGF-1 secretion in bone as a result of a burst in growth hormone secretion. IGF-1 induction could provide a survival signal that is essential during early cell transformation to circumvent EWS-FLI1-induced growth arrest and apoptosis.

## TECHNIQUES FOR DETECTION OF TRANSLOCATION

Chromosomal karyotyping is the classical method for demonstrating translocations. However, it requires fresh tumor, which needs to be cultured and highly skilled personnel to produce and interpret the karyotype. In this technique, cryptic translocations can be missed.

*In situ* hybridization (either fluorescent, i.e., FISH, chromogen, or silver based) utilizes labeled nucleic acid probes that hybridize to regions flanking the loci of interest and can detect aberrant localization of these probes. However, multiple probes would be required to detect rarer translocations. The advantage of this method is that it can be applied easily to touch preparations, fresh tissue, karyotype preparations, frozen specimens, and formalin-fixed paraffin-embedded samples.

Using polymerase chain reaction (PCR), especially reverse transcriptase PCR (RT-PCR), results in amplification of fusion transcripts encoded by specific chimeric gene. RT-PCR can be used with fresh, frozen, or formalin-fixed paraffin embedded tissue. The identity of the amplified fragment can be confirmed using multiple techniques, including DNA gel electrophoresis, restriction fragment digestion, or direct DNA sequencing.

As the formalin-fixed paraffin-embedded tissue suffers from the problems of poor primary fixation, cross-linking of nucleic acids, and heterogeneity, a combination of RT-PCR and FISH may be a better approach to enhance the sensitivity and accuracy of detecting EFT translocations.^39^

### Post-chemotherapy assessment

Excision specimens received after chemotherapy are examined thoroughly and the greatest dimension of the tumor is mapped into grids to assess necrosis. The histological response to chemotherapy is graded semiquantitatively. Grade 1 indicates 50% or less of tumor necrosis, grade 2 is more than 50% but less than 90% necrosis, grade 3 is 90–99% necrosis, and grade 4 is 100% necrosis. Patients with a good response to chemotherapy (grades 3 and 4) have superior local recurrence-free survival at 5 years (86% vs 51%, *P*=0.15).^40^ Age and sex,^41^ and the tumor size^42^ are thought to influence the degree of response to chemotherapy and event-free survival.

## FACTORS ASSOCIATED WITH PROGNOSIS

In EFT, several factors have been considered to be of prognostic importance (stage, primary tumor site, size, age, and response to therapy).^43^–^45^

Detection of metastasis is done by radiological and radionuclear scans, together with bone marrow biopsy. Many studies have tried to address the issue of occult- or micrometastasis, detected by molecular methods in the blood and bone marrow. Some investigators have found that the presence of chimeric transcripts in bone marrows of apparently nonmetastatic EFT cases at presentation can be seen in up to 43% of cases^46^ and this was associated with outcome unfavorable.^47^ Conversely, others have not shown any significant association between detection at the time of diagnosis and outcome.^46^,^48^ A significant association has been observed between increased risk of recurrence and detection of occult tumor cells by RT-PCR during follow-up (univariate analysis *P*=0.0028 and multivariate analysis *P*=0.024).^46^ In addition, some patients with more than 90% necrosis, who were positive for the chimeric transcript during follow-up, developed metastasis.

The risk of local recurrence is associated with the status of the resection margins.^42^ At the molecular level, EWS-FLI1 type 1 fusion is associated with lower proliferation rate.^49^

*p53* expression is increased in EWS-FLI1-expressing cells. The EWS-FLI1 oncoprotein is thought to abrogate the *p53* pathway, thus contributing to tumorigenesis. By univariate analysis, cases with *p53* of more than 20% have significantly poorer overall survival among patients with localized disease and in multivariate analysis, *p53*>20% is one of the strongest negative prognostic factor.^50^ *p53* mutation was noted to be the most important independent prognostic factor.^51^

The loss of Rb gene expression is very rarely described in EFT,^52^ though there was no significant correlation with metastatic disease at presentation or outcome.

Approximately 30% of EFTs show homozygous loss of *p*16, which regulates cell cycle progression.^53^ In one study, by univariate analysis, *p*16/*p*14ARF deletion alone had only marginal value as a negative factor. However, in the multivariate analysis, *p*16/*p*14ARF homozygous deletion emerged as the second most significant factor after *p53* mutation.^51^

## THERAPEUTIC TARGETS

The ultimate aim of all the research into any tumor is to find a therapeutic agent. The EWS-FLI1 fusion is present only in EFT cells and does not exist in any normal cell of the body. Thus, EFT contains a unique protein generated by tumor-specific translocation with a potential for molecular target, but so far nothing has reached the clinics. This might be due to EWS-FLI1 being a very difficult molecule to analyze directly *in vitro* due to its poor solubility.^54^

As IGF-1 is associated with EFT growth, monoclonal antibodies against this potential target are being tried.^55^ Other conceivable candidates include phospholipase D2 (PLD2)^56^ and protein tyrosine phosphatase I (PTPL1),^57^ both of which are highly expressed in EFT.

## CONCLUSION

The diagnosis of EFT amalgamates the usual or classical tools such as histology and immunohistochemistry with newer molecular technologies like FISH and PCR. The goal of these is to furnish a correct diagnosis and give sufficient information about the tumor that would aid in better risk assessment, improve clinical management, and survival of the patients.
